# Knowledge graph analysis and visualization of artificial intelligence applied in electrocardiogram

**DOI:** 10.3389/fphys.2023.1118360

**Published:** 2023-02-09

**Authors:** Mengting Yang, Hongchao Zhang, Weichao Liu, Kangle Yong, Jie Xu, Yamei Luo, Henggui Zhang

**Affiliations:** ^1^ Key Laboratory of Medical Electrophysiology, Ministry of Education and Medical Electrophysiological Key Laboratory of Sichuan Province, Collaborative Innovation Center for Prevention of Cardiovascular Diseases, Institute of Cardiovascular Research, Southwest Medical University, Luzhou, China; ^2^ School of Medical Information and Engineering, Southwest Medical University, Luzhou, China; ^3^ College of Biomedical Engineering and Instrument Science, Zhejiang University, Hangzhou, China; ^4^ School of Physical Education, Southwest Medical University, Luzhou, China; ^5^ Department of Physics and Astronomy, The University of Manchester, Manchester, United Kingdom

**Keywords:** electrocardiogram (ECG), artificial intelligence (AI), knowledge graph analysis, CiteSpace, VOSviewer

## Abstract

**Background:** Electrocardiogram (ECG) provides a straightforward and non-invasive approach for various applications, such as disease classification, biometric identification, emotion recognition, and so on. In recent years, artificial intelligence (AI) shows excellent performance and plays an increasingly important role in electrocardiogram research as well.

**Objective:** This study mainly adopts the literature on the applications of artificial intelligence in electrocardiogram research to focus on the development process through bibliometric and visual knowledge graph methods.

**Methods:** The 2,229 publications collected from the Web of Science Core Collection (WoSCC) database until 2021 are employed as the research objects, and a comprehensive metrology and visualization analysis based on CiteSpace (version 6.1. R3) and VOSviewer (version 1.6.18) platform, which were conducted to explore the co-authorship, co-occurrence and co-citation of countries/regions, institutions, authors, journals, categories, references and keywords regarding artificial intelligence applied in electrocardiogram.

**Results:** In the recent 4 years, both the annual publications and citations of artificial intelligence in electrocardiogram sharply increased. China published the most articles while Singapore had the highest ACP (average citations per article). The most productive institution and authors were Ngee Ann Polytech from Singapore and Acharya U. Rajendra from the University of Technology Sydney. The journal Computers in Biology and Medicine published the most influential publications, and the subject with the most published articles are distributed in Engineering Electrical Electronic. The evolution of research hotspots was analyzed by co-citation references’ cluster knowledge visualization domain map. In addition, deep learning, attention mechanism, data augmentation, and so on were the focuses of recent research through the co-occurrence of keywords.

## 1 Introduction

An electrocardiogram (ECG) simply records the changes in electrical activity caused by the heart and is one of the most popular used non-invasive methods for diversified biomedical applications (Alberdi et al., 2016; Kaplan Berkaya et al., 2018). The diagnosis of cardiovascular disease is the main application field of ECG analysis, especially the diagnosis of arrhythmia (Ghorbani Afkhami et al., 2016). Cardiovascular disease takes the lion’s share of death causes worldwide according to 2019 statistics from American Heart Association (Benjamin et al., 2019). It is also true that the blockage of blood vessels caused by cardiovascular disease increases the risk of stroke and myocardial infarction (Yu et al., 2015). It is well known that artificial intelligence (AI) developed rapidly over the past decade, which can be simply understood as an effort to automate intellectual tasks normally performed by humans (Chollet, 2021). Furthermore, AI has been applied to various domains including the medical field due to its advantages of saving human resources, improving efficiency, and so on (Ramesh et al., 2004). Similarly, the application of AI combined with electrocardiograms attracts more attention than ever before. The potential of AI applied in ECG is tremendous (Krittanawong et al., 2017; Hong et al., 2020). The applications of ECG for intelligent diagnosis of cardiovascular diseases have increased substantially in recent years (Andersen et al., 2019; Sharma et al., 2019; Pan et al., 2022; Yu et al., 2022). In addition, many studies on AI in ECG have been carried out such as emotion recognition especially stress level (Hwang et al., 2018; Rastgoo et al., 2019; Gedam and Paul, 2021), blood pressure (Baek et al., 2019; Li et al., 2020; Miao et al., 2020), biometric identification (Wieclaw et al., 2017; Labati et al., 2019; Srivastva et al., 2021), and so on.

Bibliometric analysis has been broadly employed in various disciplines (Li et al., 2009; Han and Ho, 2011; Zupic and Čater, 2014; Liao et al., 2018; Liu et al., 2020a), which provides more objective and reliably analyzed results based on mathematical and statistical theory to realize structured analysis (Crane, 1972). A large amount of literature information is applied in showing the knowledge graph of extant research which includes inferring research’s trends and themes over time, identifying the most prolific scholars and institutions, and so on.

In this paper, the knowledge graph analysis and visualization of the application of AI in ECG are studied for the first time and explore possible research hotspots in the future. The main contributions of this paper are listed as follows.

• Describe the overall tendencies of article publication and citation on AI applied in the ECG field, which enable researchers to know the history of AI in ECG research of the annual publication/citation and provided a valuable reference for future ECG research.
• Identify the top productive countries/regions, institutions, and authors on the research for AI applied in ECG research, which would help the scholars to know the global cooperative situation and find the potential cooperator in AI applied in ECG research.
• Present important journals and categories to facilitate researchers to find the appropriate journal and efficiently publish their articles of AI on ECG.
• Explore the cited literature to look for high-quality references, and co-citation analysis of references and keywords was adopted to explore the development process of AI in ECG research.

The remainder of the paper is organized as follows. Section 2 presents the data selection criteria and analysis methods. The knowledge graph analysis and visualization results of AI applied in ECG setup are provided in the Section 3. Section 4 explores the possibilities for future research. The Section 5 discusses the limitations of this research. Finally, a brief conclusion is given in the Section 6.

## 2 Materials and methods

### 2.1 Database

In this study, the search data source was conducted in the Web of Science Core Collection (WoSCC). WoSCC collects authoritative and influential journals in various fields. WoSCC is not only a document retrieval tool but has also become one of the most important basic evaluation tools for bibliometrics due to its strict selection criteria and citation index mechanism.

**FIGURE 1:**
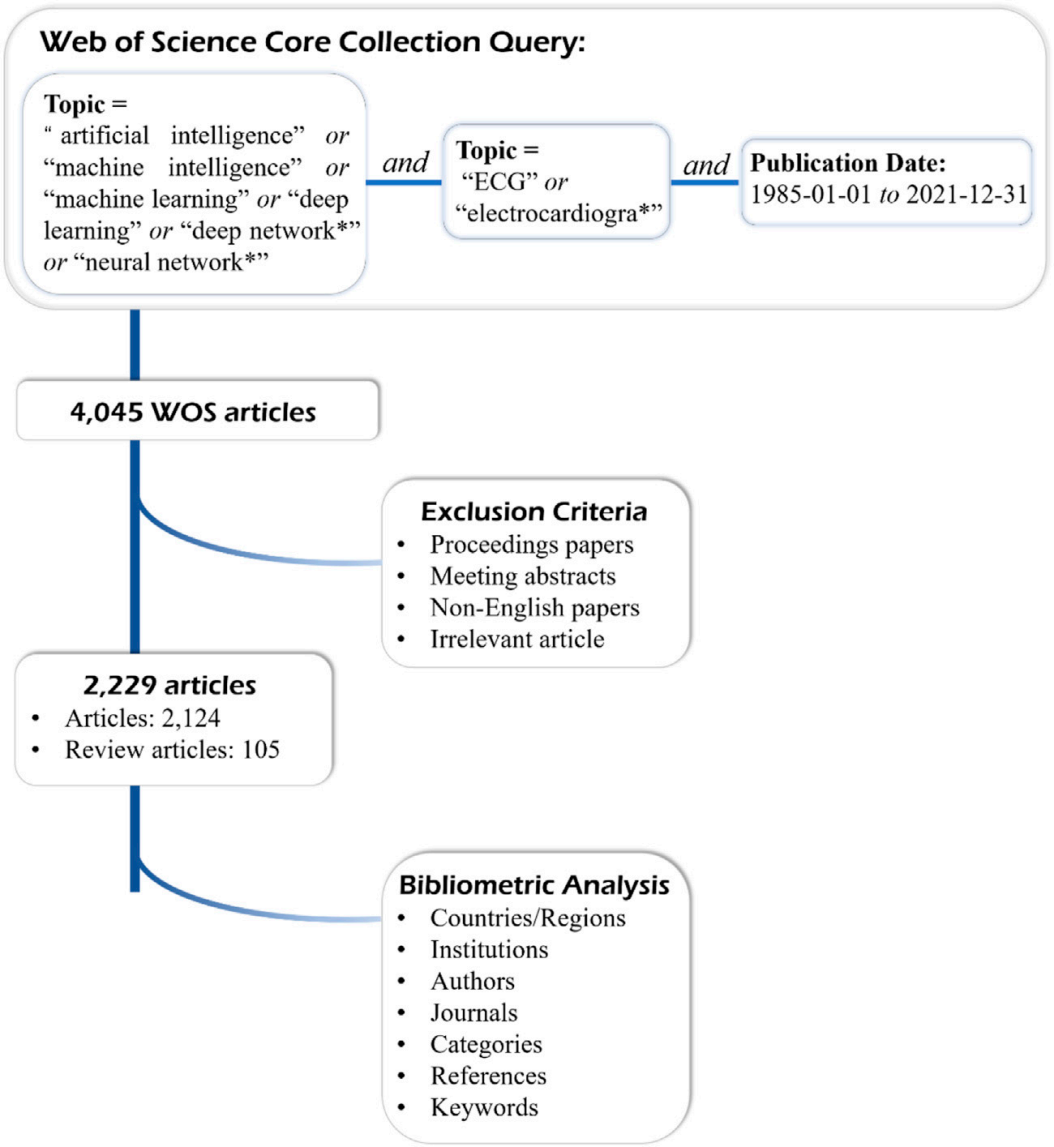
The flowchart of searching strategy and research contents.

To ensure the integrity of the research data, the AI research literature in the field of ECG was searched at all times within 1 day. The keywords of the search strategy were as follows (Figure 1): topic = (“artificial intelligence” or “machine intelligence” or “machine learning” or “deep learning” or “deep network*” or “neural network*”) and (“ECG” or “electrocardiogra*“) and publication date = (1985-01-01 to 2021-12-31). Due to the variety of branches of AI, we are concerned about a list of retrieval topics such as “machine intelligence,” “machine learning,” “deep learning,” “deep network*,” and “neural network*.” There were more than 4,000 results obtained. We discarded the documents including proceedings papers, meeting abstracts, and irrelevant articles (such as the non-electrocardiogram study is simply as ECG and so on), and the language was restricted to English. The remaining 2,229 papers are based on the above data search strategy, including 2,124 original articles and 105 review articles from WoSCC until 2021.

### 2.2 Analysis and visualization methods

The bibliometric method is a powerful and popular tool to connect heterogeneous information and get a network of relationships based on mathematical and statistical theory. It is also utilized in scientific research to analyze the knowledge graph information in a lot of different disciplines (Glenisson et al., 2005). In this paper, the bibliometric software of CiteSpace (version 6.1. R3) (Chen, 2004; Synnestvedt et al., 2005; Chen, 2006) and VOSviewer (version 1.6.18) (Van Eck and Waltman, 2007; Van Eck and Waltman, 2010) were used to analyze and visualize the co-authorship, co-occurrence of countries/regions, scholars, institutions, journals, categories, references, and keywords.

## 3 Results

### 3.1 Annual distribution analysis

**FIGURE 2:**
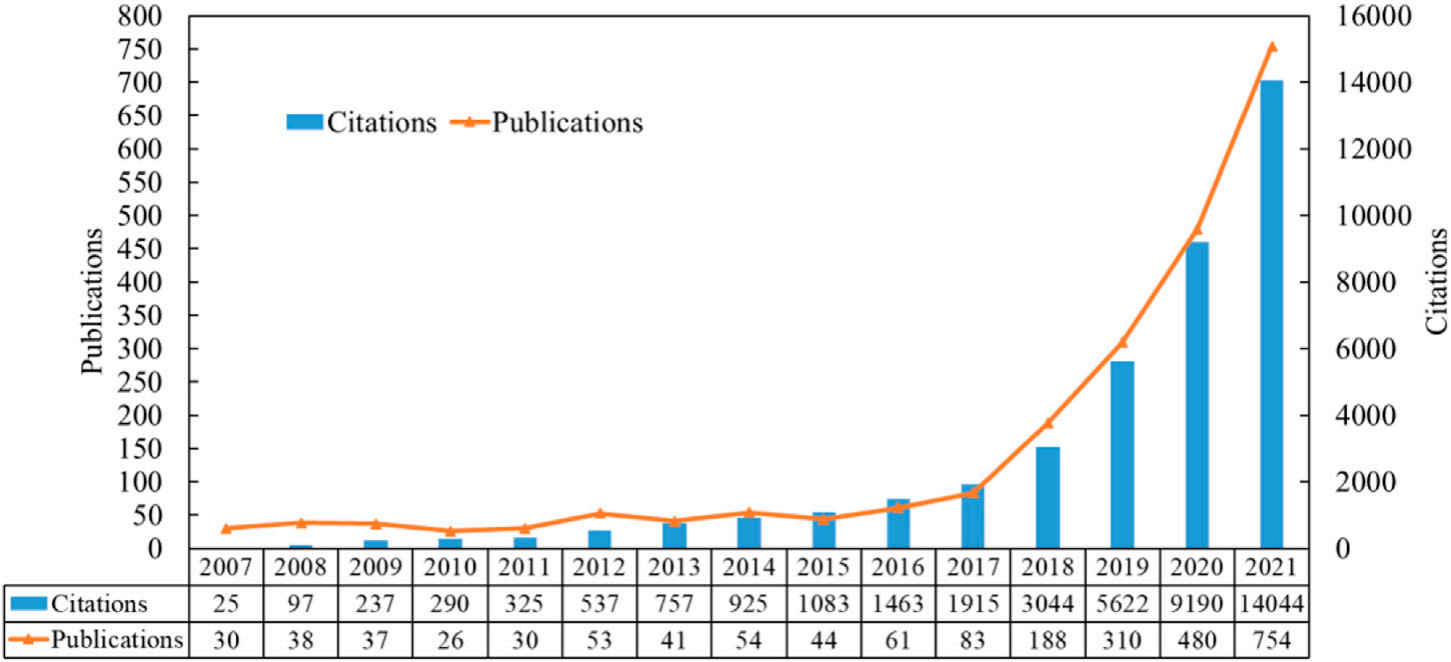
The annual distribution of publications and total citations on AI method applying in ECG research until 2021.

The chronological order distribution of literature was held to research the status of development and maturity of AI applied in the ECG field, which was shown in Figure 2. From this figure, we can observe annual quantitative publications and citation distribution of literature. The statistically analyzed results were based on the WoS analysis tool. Overall, the yearly publications presented an increased distribution with small fluctuations before 2018. However, published articles have shown a significant upward trend and developed rapidly, especially in the past 4 years, which accounted for about 78% of total publications. In addition, the citations on AI in ECG research showed an increasing trend by year, and an exponential growth trend began in 2018. In a word, no matter publications or citations, the results demonstrated that the research of AI applied in ECG entered a stage of rapid development after 2017. The characteristics of this time distribution were related to the stage of AI development. Predictably, the article count and citation number of AI in ECG will still continuously increase in the future. As for all the retrieval literature in this study, the total citation frequency was 47,371, and the H-index and average citations per document were 21.25 and 95 respectively. Therefore, the research of AI in ECG was still the focus.

### 3.2 Top productive countries/regions

**FIGURE 3:**
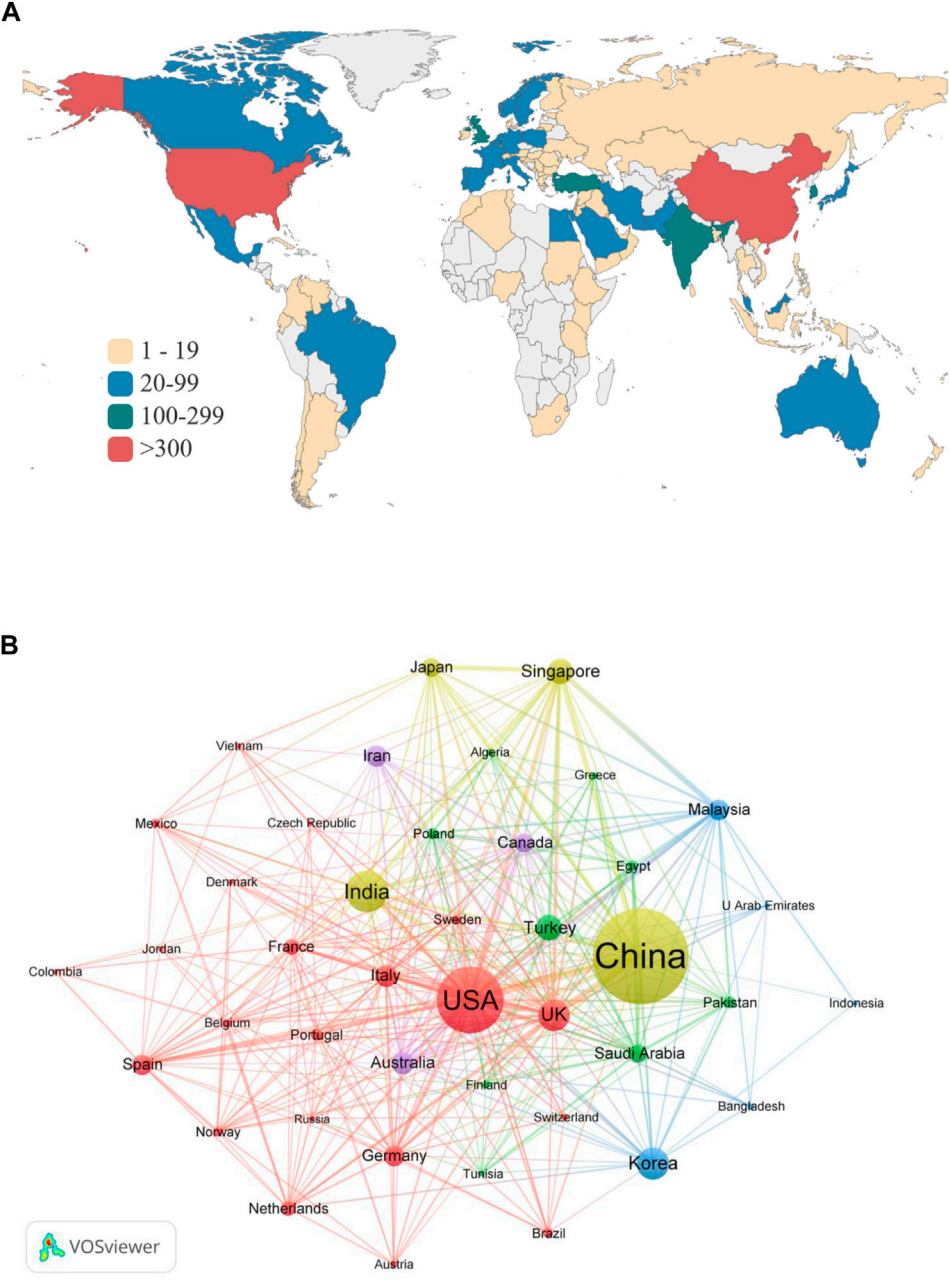
**(A)** Countries/regions’ contribution of AI applied in ECG based on total publications. **(B)** The visualization map of international co-authoring countries/regions network using VOSviewer.

Countries/regions distribution was analyzed to identify the major countries and regions involved in research on AI applied in ECG. There are 89 countries/regions in the world involved in research on AI applied in ECG research. The countries/regions distribution was shown in Figure 3A. It could be seen that China and United States published more than 300 articles on AI in ECG, and most countries and regions contributed less than 100 pieces of literature. Figure 3B illustrated the international cooperation among different countries by VOSviewer. 42 countries met the criteria when the minimum number of documents was set to more than 10. The nodes indicated different countries, and the size represented the publications for each country. The lines between nodes denoted the cooperative relationship between countries and the thickness of the lines indicated the closeness of cooperation, and then the thicker of the line represented the stronger co-authorship between countries. It can be seen that China and United States cooperated most frequently, followed by China-United Kingdom. The top 10 productive countries were summarized in Table 1. About the publications, China ranked first (710), followed by the United States (414) and India (215). What’s more, the total citations (C) and H-index (H) (Ioannidis et al., 2019) of China (11,133, 49) also ranked first, followed by the United States (10,583, 49) and Singapore (6,425, 41). In terms of the average citations per article (ACP), the top three countries were Singapore (61.78), Turkey (40.43), and Italy (27.94). The total link strength (TLS) indicated the cooperation among countries, the top three international cooperation were China (352), the United States (340), and Singapore (222).

**TABLE 1:** Top 10 productive countries related to AI on ECG.

Rank	Country	A	C	ACP	H	TLS
1	China	710	11,133	15.68	49	352
2	United States	414	10,583	25.56	49	340
3	India	215	4,214	19.60	34	119
4	Republic of Korea	150	2,238	14.92	27	55
5	United Kingdom	149	3,791	25.44	34	213
6	Türkiye	108	4,366	40.43	32	63
7	Singapore	104	6,425	61.78	41	222
8	Italy	89	2,487	27.94	24	83
9	Australia	86	1,730	20.12	20	110
10	Iran	83	1,823	21.96	24	40

A, article count; C, citation count; ACP, average citations per article; H h-index; TLS, total link strength.

### 3.3 Contribution of top organizations

**FIGURE 4:**
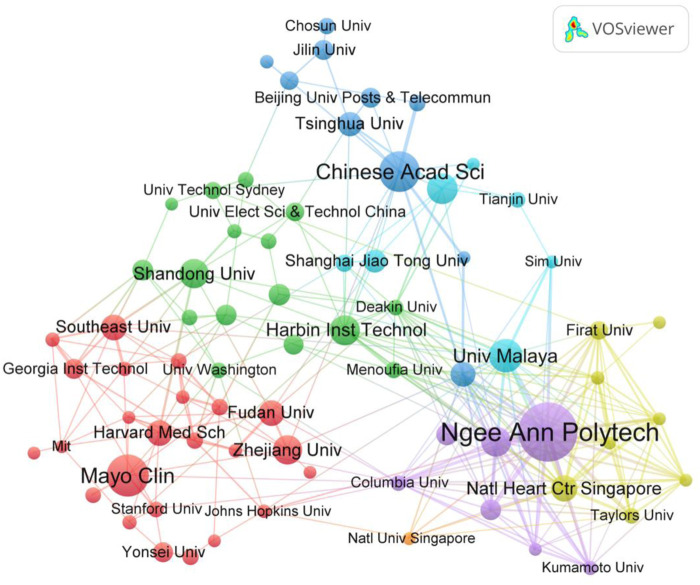
The visualization map of international co-authoring institutions network using VOSviewer.

A total of 2,630 organizations have published related articles in the field of AI applied in ECG. To discover the main research productive organizations and the co-authorship between institutions, we plotted the visualization map of the co-authorship network using VOSviewer as shown in Figure 4. There were 75 institutions that the minimum number of documents of an organization was more than 10. It could be found that Ngee Ann Polytech from Singapore had the most literature (74), Mayo Clinic ranked second (47), followed by the Chinese Academy of Sciences (45) in Table 2. About ACP, the top three were: Universiti Malaya, National Heart Centre Singapore, and Ngee Ann Polytech. The ACP of institutions from Singapore ranked top, indicating that the quality of its publications was much higher than in other countries. Regarding TLS, ranked three top institutions were Ngee Ann Polytech (209), National Heart Centre Singapore (101), and Asia University (100). Among the top 10 institutions in terms of publications, institutions from China accounted for half of the total. However, research institutions from China lacked cooperation and the influence of articles.

**TABLE 2:** Top 10 productive institutions concerning the research of AI on ECG.

Rank	Institutions	A	C	ACP	TLS	Country
1	Ngee Ann Polytech	74	5,642	76.24	209	Singapore
2	Mayo Clinic	47	1281	27.26	12	United States
3	Chinese Academy of Sciences	45	886	19.69	38	China
4	Universiti Malaya	35	3,857	110.20	63	Malaysia
5	Zhengzhou University	31	187	6.03	22	China
6	Harbin Institute of Technology	30	714	23.80	27	China
7	Asia University	30	396	13.20	100	Japan
8	Shandong University	29	737	25.41	21	China
9	Zhejiang University	29	389	13.41	10	China
10	National Heart Centre Singapore	26	2077	79.88	101	Singapore

A, article count; C, citation count; ACP, average citations per article; TLS, total link strength.

### 3.4 Contributions of authors

**FIGURE 5:**
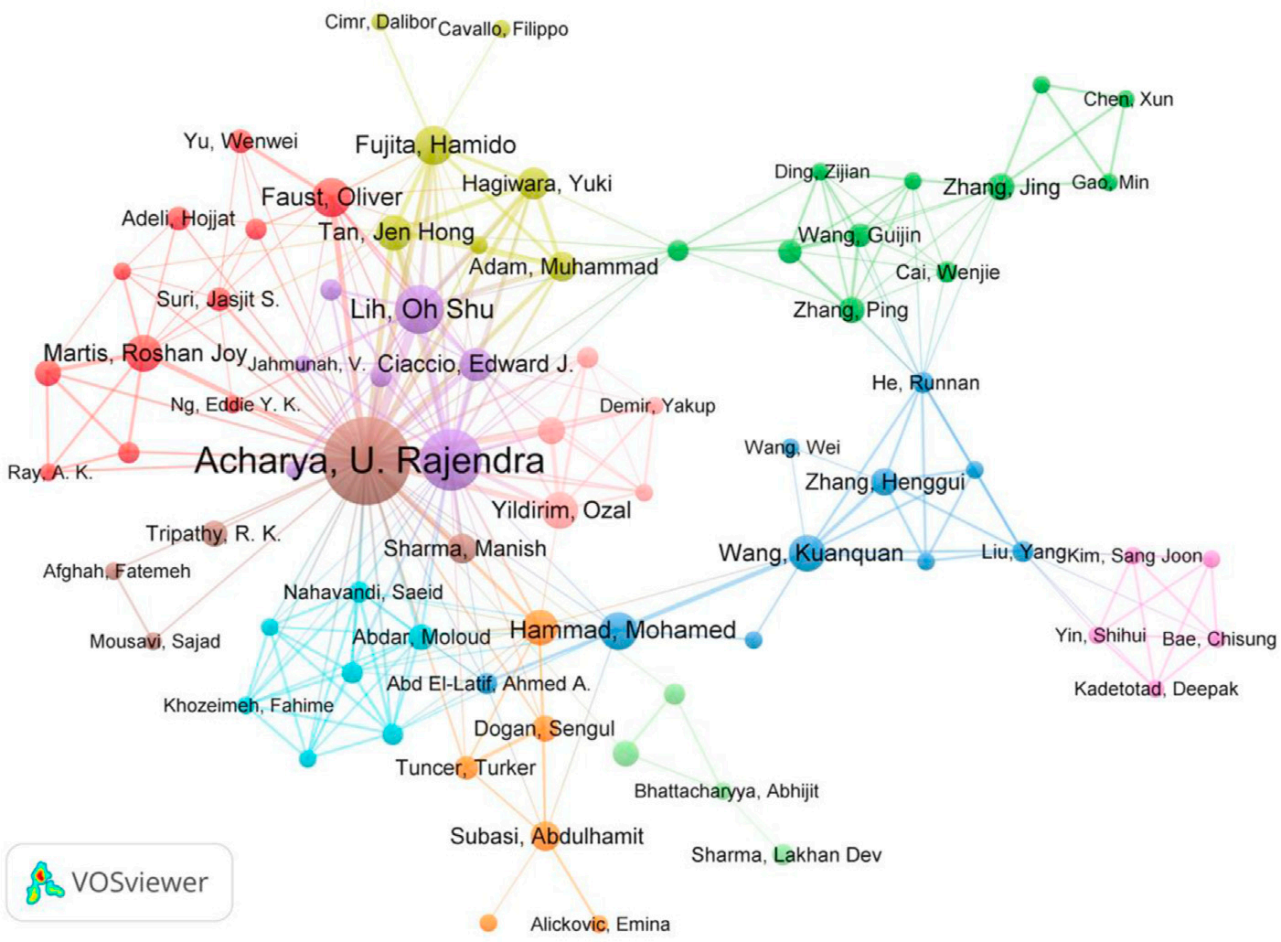
The visualization map of international co-authoring authors network using VOSviewer.

More than 8,370 authors participated in the research of AI in ECG. The visualization map of 574 authors’ cooperation when an authors’ minimum number of documents of was limited as three is shown in Figure 5. In addition, Table 3 summarizes the top 10 productive authors. Acharya U. Rajendra from the University of Technology Sydney, Tan Ru San from National Heart Centre Singapore, and Friedman Pau A. from Mayo Clinic ranked top three productive authors with 68, 33, and 31 articles respectively. Their articles also have a higher citation, H-index, and ACP. Lih Oh Shu from Ngee Ann Polytechn published 21 articles and ranked 6, but the ACP (129.38) was the highest, and then his publications are worthy of highlighting and paying more attention to this field.

**TABLE 3:** Top 10 productive authors.

Rank	Author	A	C	ACP	H	Institution
1	Acharya U. Rajendra	68	5,607	82.46	39	University of Technology Sydney
2	Tan Ru San	33	2,184	66.18	16	National Heart Centre Singapore
3	Friedman Pau A.	31	1070	34.52	14	Mayo Clinic
4	Noseworthy Peter A.	30	1076	35.87	14	Mayo Clinic
5	Attia Zachi I.	25	1036	41.44	14	Mayo Clinic
6	Lih Oh Shu	21	2,717	129.38	13	Ngee Ann Polytechn
7	Lopez-jimenez Francisco	21	852	40.57	9	Mayo Clinic
8	Kapa Suraj	20	838	41.90	8	Mayo Clinic
9	Liu Chengyu	16	373	23.31	9	Chinese Academy of Medical Sciences—Peking Union Medical College
10	Ubeyli Elif Derya	15	576	38.40	11	TOBB Ekonomi ve Teknoloji University

A, article count; C, citation count; ACP, average citations per article; H, h-index.

### 3.5 Analysis of category and top journals

**FIGURE 6:**
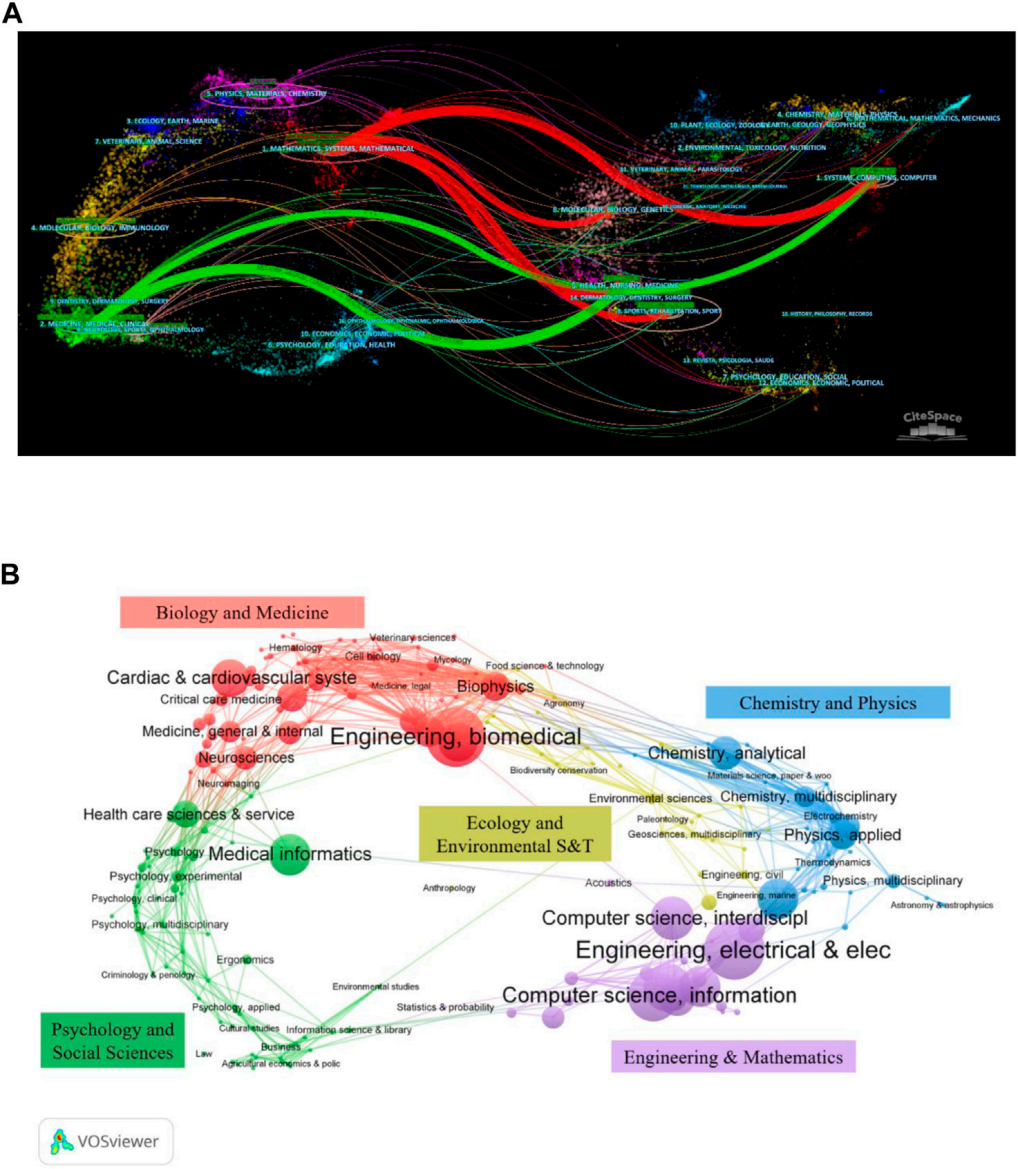
**(A)** The dual overlap map of journals on AI in ECG by using CiteSpace. **(B)** The subject category distribution on AI research in ECG was carried out by VOSviewer.

The analyzed journal distribution is adopted to identify the crucial journals in this field. The retrieved 2,229 documents were published in 469 journals, of which 41 journals were more than 10 publications. Table 4 lists the top 10 productive journals, three journals with no less than 100 articles were the most productive journal IEEE Access with 146 articles, followed by Sensors (119) and Biomedical Signal Processing (112). Furthermore, there were four journals from the United Kingdom, three from the United States, two from Switzerland, and one journal from the Netherlands. In terms of the 2021 Journal Citation Report (JCR 2021), there were four journals each located in Q1 and Q2 among the top 10 productive journals. Table 4 also shows that the partition of the journals is positively correlated with the ACP index, and the Q1 journals correspond to a higher ACP than the magazines in other partitions. The dual map is adopted to identify the relationship between citing journals (left) and cited journals (right) as demonstrated in Figure 6A. It can be seen that there were six thick citation lines, and the citing literature is mainly located in two fields: 1) Medicine, Medical, Clinical; 2) Mathematics, Systems, Mathematical. On the contrary, the cited publications primarily distributed in four fields: 1) Health, Nursing, Medicine; 2) Systems, Computing, Computer; 3) Molecular, Biology, Genetics; 4) Sports, Rehabilitation, Sport.

**TABLE 4:** Top 10 productive journals and WOS category.

Rank	Journal title	A	ACP	Country	JCR (2021)	WOS category	A
1	IEEE Access	145	15.17	United States	Q2	Engineering Electrical Electronic	557
2	Sensors	119	11.64	Switzerland	Q2	Engineering Biomedical	534
3	Biomedical Signal Processing and Control	112	23.54	United Kingdom	Q2	Computer Science Information Systems	347
4	Computers in Biology and Medicine	73	46.88	United States	Q1	Computer Science Artificial Intelligence	309
5	Computer Methods and Programs in Biomedicine	69	37.49	Netherlands	Q1	Computer Science Interdisciplinary Applications	245
6	Physiological Measurement	63	18.40	United Kingdom	Q3	Medical Informatics	220
7	Expert Systems with Applications	46	54.24	United Kingdom	Q1	Telecommunications	204
8	Applied Sciences Basel	40	8.83	Switzerland	Q3	Instruments Instrumentation	200
9	IEEE Journal of Biomedical and Health Informatics	37	26.00	United States	Q1	Mathematical Computational Biology	181
10	Scientific Reports	34	13.32	United Kingdom	Q2	Cardiac Cardiovascular Systems	176

A, article count; C citation count; ACP, average citations per article.

In the light of the WOS category, the 2,229 articles concerning AI in ECG were concentrated in 109 subjects, among which the top 10 productive were shown in the last two columns of Table 4. The superposition graph analysis of the application of AI in ECG was carried out in the field dimension, as shown in Figure 6B. It can be obtained that the top three fields of paper production were Engineering Electrical Electronic (557), Engineering Biomedical (534), and Computer Science Information Systems (347) respectively.

### 3.6 Top cited literature and co-citation analysis of references

To explore the most influential literature in the field of AI applied in ECG, there were 2,229 documents collected in this study, of which 55 publications have been cited more than 100 times. The top 10 articles with the highest citation are listed in Table 5, of which nine articles were published from 2013 to 2019, three of the most cited articles in 2016 (Kiranyaz et al., 2016; Luz et al., 2016; Rahhal et al., 2016), and one article authored by Ince et al. (2009) published in 2009. Only one paper was a single author Yildirim (2018), and the other nine articles were co-authored. The top three cited papers were published by Hannun et al. (2019), Kiranyaz et al. (2016), and Acharya et al. (2017a). Luz et al. (2016) show the highest total link strength.

**TABLE 5:** Top 10 citing references related to AI on ECG.

Rank	Title	Author	Journal	C	TLS	Year
1	Cardiologist-level arrhythmia detection and classification in ambulatory electrocardiograms using a deep neural network	Hannun AY	Nature Medicine	738	13	2019
2	Real-time patient-specific ECG classification by 1-d convolutional neural networks	Kiranyaz S	IEEE Transactions on Biomedical Engineering	732	33	2016
3	A deep convolutional neural network model to classify heartbeats	Acharya UR	Computers in Biology and Medicine	451	33	2017
4	Deep learning for healthcare applications based on physiological signals: a review	Faust O	Computer Methods and Programs in Biomedicine	374	15	2018
5	ECG beat classification using PCA, LDA, ICA and Discrete Wavelet Transform	Martis RJ	Biomedical Signal Processing and Control	356	29	2013
6	ECG-based heartbeat classification for arrhythmia detection: A survey	Luz EJD	Computer Methods and Programs in Biomedicine	351	39	2016
7	Application of deep convolutional neural network for automated detection of myocardial infarction using ECG signals	Acharya UR	Information Sciences	350	29	2017
8	Deep learning approach for active classification of electrocardiogram signals	Al Rahhal MM	Information Sciences	302	29	2016
9	A Generic and Robust System for Automated Patient-Specific Classification of ECG Signals	Ince T	IEEE Transactions on Biomedical Engineering	272	31	2009
10	A novel wavelet sequence based on deep bidirectional LSTM network model for ECG signal classification	Yildirim O	Computers in Biology and Medicine	270	35	2018

C, citation count; TLS, total link strength.

**FIGURE 7:**
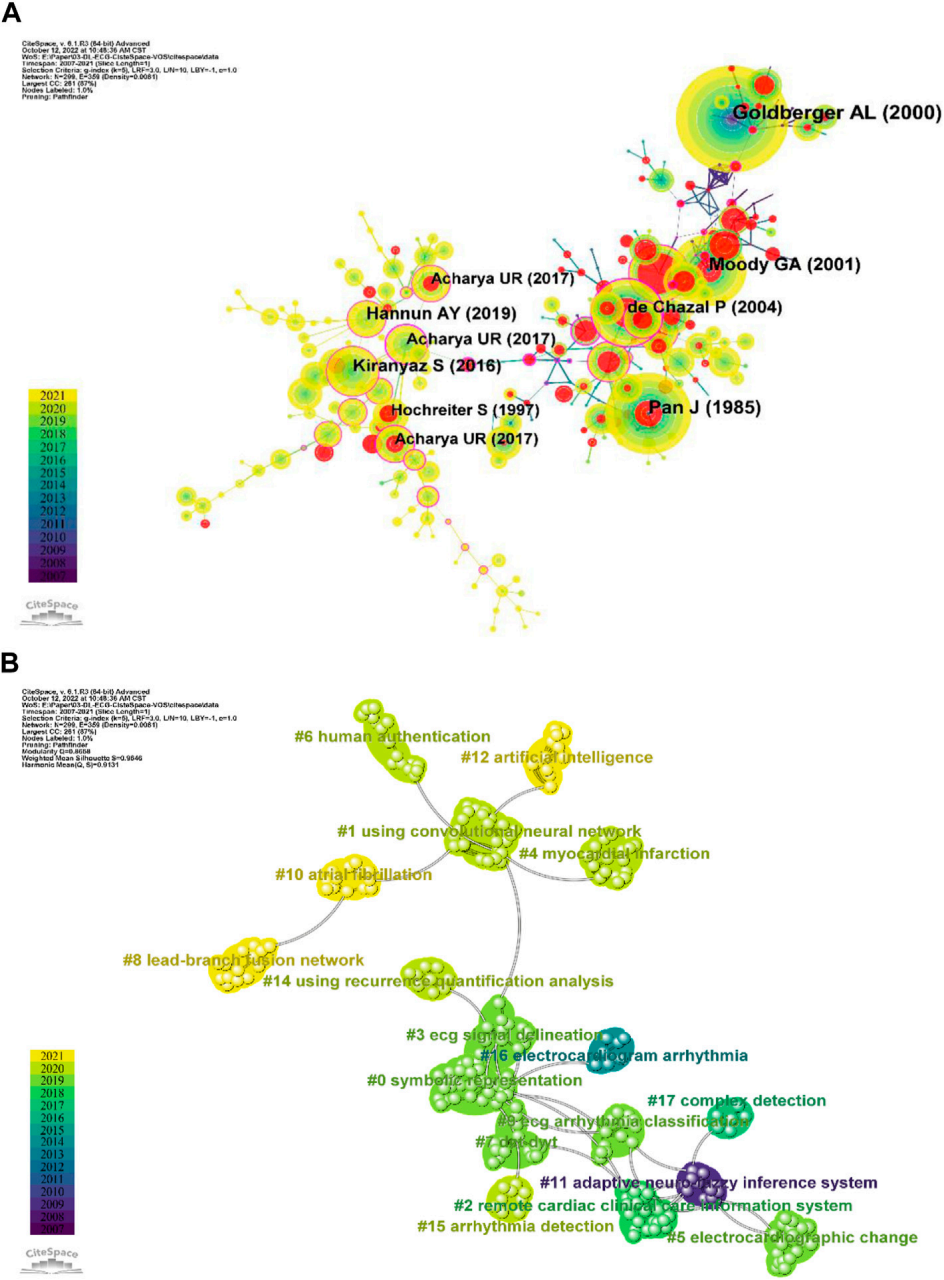
**(A)** The CiteSpace co-citation knowledge domain map for references changing over the years. The frequency of citations is indicated by different node sizes, red circle indicates a burst of cited references. **(B)** Clustering knowledge visualization domain map for co-citation references over time in CiteSpace. The evolution of research hotspots is shown with different colored nodes.

The co-citation of references was analyzed to map the knowledge domain and the knowledge base of AI in ECG (Chen, 2004). A total of 52,379 cited references were included in this study. Table 6 summarizes the top 10 with the highest cited references. Goldberger et al. (2000) had the highest total citation frequency and total link strength, with 601 citations and 1448 TLS, followed by Pan and Tompkins (1985) with 391 citations and Moody and Mark (2001) with 344 citations. There are two articles from Acharya et al. (2017a) and Acharya et al. (2017b) appearing in both the most cited references and citing papers. We used CiteSpace to explore the co-citation knowledge domain map, as shown in Figure 7A, which visually shows the top 10 with the highest cited references changing over the years. The nodes represent different cited articles, the bigger node indicates higher citations, and the results are consistent with Table 6. The burst was marked by the red circle, where the larger the radius means a bigger burst value, which means that the citations of this document vary greatly in a short period. Notably, the distribution of bursts and citations are shown as different tendencies, future discussion will be elaborated on in more detail in the discussion section. Figure 7B shows the clustering knowledge graph over time, visually showing the changing trend of research hotspots over time. The changes in clusters and labels are identified by colors representing different years. It is important to note that, the number of clusters is independent of time and sorted based on the number of references included. The Harmonic Mean (Q, S) was 0.9131 (Modularity Q = 0.8668, Weighted Mean Silhouette S = 0.9646), which indicates good clustering and higher homogeneity of the clustering network.

**TABLE 6:** Top 10 co-cited references related to AI on ECG.

Rank	Title	Author	Journal	C	TLS	Year
1	PhysioBank, PhysioToolkit, and PhysioNet: Components of a New Research Resource for Complex Physiologic Signals	Goldberger AL	Circulation	601	1448	2000
2	A Real-Time QRS Detection Algorithm	Pan J	IEEE Transactions on Biomedical Engineering	391	1025	1985
3	The impact of the MIT-BIH Arrhythmia Database	Moody GA	IEEE Transactions on Biomedical Engineering	344	1265	2001
4	Real-Time Patient-Specific ECG Classification by 1-D Convolutional Neural Networks	Kiranyaz S	IEEE Transactions on Biomedical Engineering	268	1204	2016
5	Automatic classification of heartbeats using ECG morphology and heartbeat interval features	de Chazal P	IEEE Transactions on Biomedical Engineering	237	1096	2004
6	Cardiologist-level arrhythmia detection and classification in ambulatory electrocardiograms using a deep neural network	Hannun AY	Nature Medicine	223	707	2019
7	A deep convolutional neural network model to classify heartbeats	Acharya UR	Computers in Biology and Medicine	184	946	2017
8	Automated detection of arrhythmias using different intervals of tachycardia ECG segments with convolutional neural network	Acharya UR	Inform Sciences	153	589	2017
9	Application of deep convolutional neural network for automated detection of myocardial infarction using ECG signals	Acharya UR	Inform Sciences	152	726	2017
10	Long Short-Term Memory	Hochreiter S	Neural Comput	148	502	1997

C, citation count; TLS, total link strength.

### 3.7 Keywords co-occurrence analysis

**FIGURE 8:**
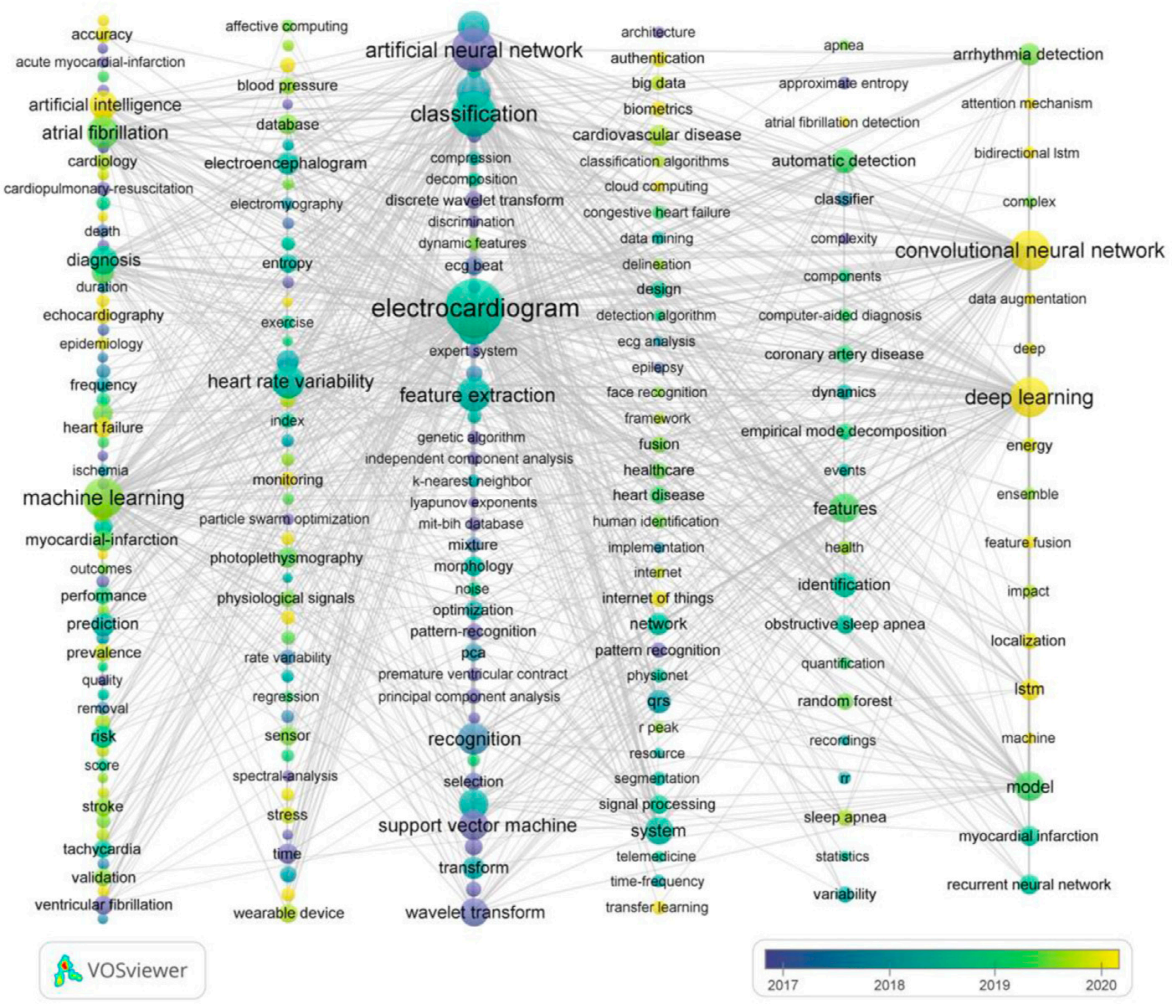
The overlay co-occurrence visualization network of 229 keywords with a frequency of more than 10 times for AI in ECG by using VOSviewer. Different colors of nodes represent the average year in which keywords appear.

The co-occurrence of keywords was adopted to identify its research frontiers over time. A total of 6,409 keywords were included in this study, of which 229 keywords with a frequency of more than 10 occurrences. Table 7 lists the top 20 keywords with the highest frequency, of which the “Electrocardiogram” with 1224 O (occurrences) and 6383 TLS (total link strength), followed by “Classification” (559 O, 3321 TLS) and “Artificial neural network” (497 O, 2582 TLS). VOSviewer was used to analyze the knowledge domain map of keywords co-occurrence cluster and generated the overlay visualization network as illustrated in Figure 8, which identified the change of keywords over time. The color of nodes represents the average time of keywords occurrence. The yellow nodes showed the latest research hotspot keywords. It is obvious that “convolutional neural network,” “deep learning,” “attention mechanism,” “data augmentation,” “cloud computing,” “internet of things,” “echocardiography” and so on were keywords that had appeared frequently in recent years, indicating that they may be hotspots for future research.

**TABLE 7:** Top 20 co-occurrence keywords.

Rank	Keyword	O	TLS	Rank	Keyword	O	TLS
1	Electrocardiogram	1224	6,383	11	Atrial fibrillation	212	1136
2	Classification	559	3,321	12	Algorithm	209	1279
3	Artificial neural network	497	2,582	13	Recognition	194	1197
4	Deep learning	409	2,218	14	Support vector machine	164	1001
5	Machine learning	406	2,116	15	Model	158	956
6	Convolutional neural network	378	2,114	16	Signal	156	983
7	Cardiac arrhythmias	253	1535	17	Features	155	1051
8	Feature extraction	252	1712	18	Diagnosis	150	998
9	Heart rate variability	222	1189	19	Artificial intelligence	148	754
10	Electrocardiogram classification	212	1190	20	System	142	844

O, occurrences count; TLS, total link strength.

## 4 Discussion

In this study, VOSviewer and CiteSpace widely used software is first adopted to do comprehensive bibliometrics analysis through using retrieved literature for AI in ECG research, which is much more essential to reveal the development trend of research and predict the hotspots for future research (Merigó and Yang, 2017).

The publications and citations of literature generally showed an upward trend, and there can be divided into two stages: 1) the primary stage (2007–2016): The number of articles published annually showed a fluctuating trend, all of which did not exceed 70, although the number of citations increased slowly year by year. 2) the rapid development stage (2017- now): 83 papers were published in 2017, and the number of papers increased rapidly every year, reaching 754 in 2021.

More than half of the countries published less than 10 in this study. Although the publications from China accounted for about one-third of the total, the ACP (average citations per article) was only 15.69, Singapore and Turkey ranked first (61.78 ACP) and second (40.43 ACP), which revealed that although China had a large number of articles published, it lacked high-quality papers for AI applied in ECG. A similar conclusion can be drawn about productive institutions, the ACP from Chinese institutions was comparatively low while those from Singapore were relatively high. The possible reason may be that communication with high-level international institutions was limited due to language barriers for Chinese authors, which leads to a little less innovation and lower citations of published articles from Chinese authors. In addition, the strong cooperation among authors from Singapore (as shown in Table 2) promotes the quality of publications. Half of the top 10 most prolific authors came from Mayo Clinic in the United States, and two from Singapore. The H-index of Acharya U. Rajendra from the University of Technology Sydney ranked first and was 39, which meant his high level of academic output in this research field. It is worth noting due to the limitations of VOSviewer and CiteSpace, the author’s citation counts include self-citation counts. Although the final results were not affected by a review of the top ten authors, warranting further investigation in future studies.

Figure 6A illustrated the dual overlap map of journals on AI in ECG, which was a method to display the distribution and citation tracks of papers in various disciplines (Chen and Leydesdorff, 2014). The vertical axis of the ellipse represented the number of articles published, while the horizontal axis denoted the number of authors. In terms of citing journals, the largest number of authors was in the field of Physics, Materials, Chemistry. The Sports, Rehabilitation, Sport field was the most cited article. Supplementary Table S1 showed the six most prominent reference paths and the specific parameters (z-score and f), which demonstrated the intersectionality of references from different domains.

The network of co-citation references as shown in Figure 7B consisted of 17 clusters and were labeled by LLR (Chen et al., 2010). The largest cluster (#0) has 32 cited references and was labeled as “symbolic representation,” the major citing article is Xie et al. (2020), which systematically reviewed the application of computational diagnostic techniques including classic machine learning and end-to-end deep learning algorithms based on ECG signals to estimating cardiovascular diseases (CVDs) conditions. The most cited members in this cluster were Kiranyaz et al. (2016), Acharya et al. (2017a) and Acharya et al. (2017c). The earliest cluster was the “adaptive neuro-fuzzy inference system” (#11) in this research included 12 members, in which most of the articles were cited by Elif (Ubeyli, 2009). “Lead-branch fusion network” (#8), “atrial fibrillation” (#10), and “artificial intelligence” (#12) were the latest research hotspots, which included 14, 13, and 12 members respectively. For “artificial intelligence” (#12) in this study, the mean year of occurrence was 2018. This topic was first cited in 2016 (He et al., 2016) and continued to 2020 (Ko et al., 2020; Raghunath et al., 2020; Saadatnejad et al., 2020), indicating that deep learning was focused on by more and more researchers of AI in ECG.

Supplementary Figure S1 visually shows the top 25 references with the strongest citation bursts. The ECG beat recognition using a fuzzy hybrid neural network put forward by Osowski and Linh (2001) gave the greatest strength, which began from 2007 to 2014. A deep residual learning framework presented by He et al. (2016) eased the training of networks and made it substantially deeper than those used previously, its excellent performance made it still frequently cited from 2019 to 2021. Perekrestenko et al. (2017) proposed prominent coordinate descent methods that employed random partial updates of decision variables to solve huge-scale convex optimization problems, which was the same as He et al. (2016) frequent citations in the last 4 years.

We identified the changing trend of keywords over time as shown in Figure 8, which to better capture the changes in research hotspots. However, due to the built-in algorithm of VOSviewer (Eck and Waltman, 2009; Van Eck and Waltman, 2010), the specific content of AI applied in ECG failed to exactly divide based on expectations, which warrants further study in the future.

## 5 Limitations

In this study, the latest studies on AI-assisted ECG applications have been analyzed from a knowledge graph perspective to disclose the hotspots in this area. However, for some research hotspots, such as a general outline of the development was shown on the development of clinical application scenarios and so on, while its details were not presented visually. AI-assisted ECG research has been applied in different types of cardiac and cardiovascular diseases including myocardial ischemia (Zhao et al., 2022) and arrhythmias (Lee et al., 2021), which showed the potential to be combined with computational methods, e.g., computational fluid dynamics modeling (Liu et al., 2020b) and electromechanical simulation (Zang et al., 2011). The evaluation of AI-assisted ECG analysis methods in different types of diseases, different application scenarios (i.e., in-hospital or daily monitoring), and different cohorts deserve further investigation.

In addition, the bibliometric analysis was adopted on English literature from WoSCC in this study, which may lead to the omission of information from publications by non-English language on AI applied in the ECG research field. Furthermore, article citations (including self-citation) change over time, and the literature data used in this study can only present the research status at the time of data collection, while the research hotspots and content cannot be tracked in real-time.

## 6 Conclusion

In recent years, the number of articles published and citations on the application of AI in ECG has been continuously increasing, and it will continue to maintain the trend of growth in the future. On the whole, China had the most but lower high-quality articles while Singapore had the uppermost quality of documents on AI in ECG due to the highest ACP and international cooperation. Although Chinese institutions accounted for half of the top 10 publications, the overall cooperation intensity was weak, with Ngee Ann Polytech from Singapore having the strongest cooperation. Therefore, cooperative communication among institutions should be strengthened, especially in China and the United States (top two publications). It should be noted that China has only one prolific author while American authors account for half of the top 10 productive authors. Acharya U. Rajendra from the University of Technology Sydney ranked first in the number of published papers, far higher than other authors, so his publications deserve special attention. In terms of the distribution of published journals, the ACP index of the journal is consistent with the JCR partition. The ACP of Expert Systems with Applications from the United Kingdom has the highest index but ranks seventh in the top 10 productive journals. The category of Engineering Electrical Electronic and Engineering Biomedical ranked first and second respectively in terms of the number of publications. The AI method of ECG has also changed from a support vector machine at the beginning to the current popular deep learning. The direction of studies through ECG has also evolved from the initial ventricular fibrillation to focusing on a variety of other subjects (blood pressure, stress, biometric identification, sleep apnea, etc.).

## Data Availability

The original contributions presented in the study are included in the article/Supplementary Material, further inquiries can be directed to the corresponding author.
