# Supplementary figures and images for: Knowledge graph analysis and visualization of artificial intelligence applied in electrocardiogram
Source: Front Physiol. 2023 Feb 9;14:1118360. doi: 10.3389/fphys.2023.1118360 (PMC9947408; doi:10.3389/fphys.2023.1118360)

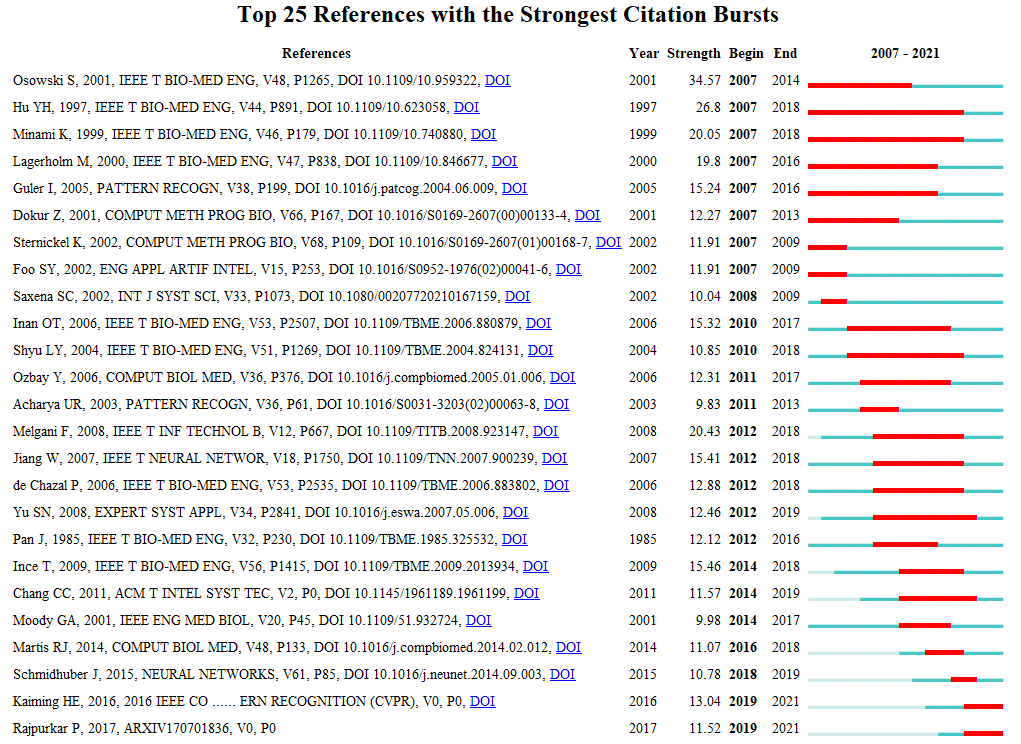

Supplement: Supplementary file 1 [file Image1.JPEG]

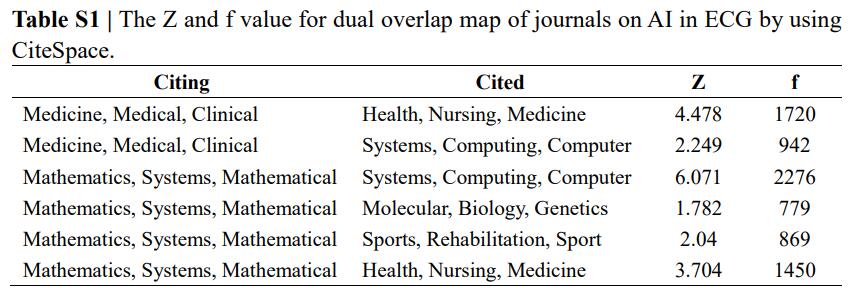

Supplement: Supplementary file 2 [file Image2.JPEG]
